# Attenuation of Quorum Sensing Regulated Virulence of *Pectobacterium carotovorum* subsp. *carotovorum* through an AHL Lactonase Produced by *Lysinibacillus* sp. Gs50

**DOI:** 10.1371/journal.pone.0167344

**Published:** 2016-12-02

**Authors:** Sneha S. Garge, Anuradha S. Nerurkar

**Affiliations:** Department of Microbiology and Biotechnology Centre, Faculty of Science, The Maharaja Sayajirao University of Baroda, Vadodara, Gujarat, India; Agricultural University of Athens, GREECE

## Abstract

Quorum sensing (QS) is a mechanism in which Gram negative bacterial pathogens sense their population density through acyl homoserine lactones (AHLs) and regulate the expression of virulence factors. Enzymatic degradation of AHLs by lactonases, known as quorum quenching (QQ), is thus a potential strategy for attenuating QS regulated bacterial infections. We characterised the QQ activity of soil isolate *Lysinibacillus* sp. Gs50 and explored its potential for controlling bacterial soft rot of crop plants. *Lysinibacillus* sp. Gs50 inactivated AHL, which could be restored upon acidification, suggested that inactivation was due to the lactone ring hydrolysis of AHL. Heterologous expression of cloned gene for putative hydrolase (792 bp) designated *adeH* from *Lysinibacillus* sp. Gs50 produced a ~29 kDa protein which degraded AHLs of varying chain length. Mass spectrometry analysis of AdeH enzymatic reaction product revealed that AdeH hydrolyses the lactone ring of AHL and hence is an AHL lactonase. Multiple sequence alignment of the amino acid sequence of AdeH showed that it belongs to the metallo- β- lactamase superfamily, has a conserved “HXHXDH” motif typical of AHL lactonases. *K*_*M*_ for AdeH for C6HSL was found to be 3.089 μM and the specific activity was 0.8 picomol min^-1^μg^-1^. AdeH has not so far been reported from any *Lysinibacillus* sp. and has less than 40% identity with known AHL lactonases. Finally we found that *Lysinibacillus* sp. Gs50 can degrade AHL produced by *Pectobacterium carotovorum* subsp. *carotovorum* (Pcc), a common cause of soft rot. This QQ activity causes a decrease in production of plant cell wall degrading enzymes of Pcc and attenuates symptoms of soft rot in experimental infection of potato, carrot and cucumber. Our results demonstrate the potential of *Lysinibacillus* sp. Gs50 as a preventive and curative biocontrol agent.

## Introduction

Many bacteria communicate with each other and respond collectively to a changing environment. They do this by using a cell-to-cell communication mechanism known as quorum sensing (QS), in which bacteria secret diffusible signal molecules and respond to the accumulation of signal in the environment in a cell density-dependent manner [1]. QS therefore allows the synchronous expression of target genes by a bacterial community [2, 3]. Many human and plant pathogenic Gram negative bacteria (including *Agrobacterium*, *Brucella*, *Burkholderia*, *Erwinia*, *Enterobacter*, *Pseudomonas*, *Ralstonia*, *Serratia*, *Vibrio* and *Yersinia* sp.) use *N*- acyl homoserine lactones (AHLs) as QS signals to regulate the expression of virulence factors [4, 5]. With increasing population density of bacteria more AHL accumulates in the environment and achieves its critical threshold concentration. AHL binds to and activates its cognate transcriptional regulator to trigger the expression of target genes [6]. For example, the plant pathogen *Pectobacterium carotovorum* subsp. *carotovorum* (Pcc) primarily uses 3-oxo-hexanoyl homoserine lactone (3OC6HSL) as its QS signal. This chiefly controls the expression of secretory plant cell wall degrading enzymes (pectate lyases, protease and cellulase) that macerate plant tissues and contribute to the soft rot phenotype [7, 8]. Some of the Pcc strains utilise 3-oxo-octanoyl homoserine lactone (3OC8HSL) to a lesser extent for the same purpose. Because QS regulates the amount of damage pathogens can cause to their host, it has been suggested that disrupting QS could constitute a new approach to reduce infection and so control soft rot and other infections.

Interference with QS is termed as quorum quenching (QQ) and can be achieved in many different ways. The production of signal molecules, the signal molecule itself, and/or the sensing of the signal molecule by the cognate regulatory protein could all be targets for QQ. The molecules used to inhibit QS could be of abiotic or biotic origins, but include enzymes of bacterial origin that degrade QS signals [9, 10]. The majority of QQ enzymes of bacterial origin are either AHL lactonases or AHL acylases. AHL lactonases catalyse hydrolytic opening of the lactone ring in AHL molecule to form N-acyl homoserine as product. This kind of hydrolysis can be reversed at acidic pH and may also occur spontaneously at alkaline pH [11]. The first reported QQ enzyme was an AHL lactonase (AiiA) from a Gram-positive Firmicute *Bacillus* sp. 240B1 [12]. Subsequently, other bacteria that produce AHL lactonase were found among the Firmicutes (e.g. *B*. *thuringiensis*, *B*. *cereus*, *B*. *anthracis*, *B*. *mycoides*, *B*. *subtilis*, *B*. *amyloliquefaciens*, *Geobacillus* sp.) [13–18]; and also in the Actinobacteria (*Arthrobacter sp*., *Microbacterium testaceum*, *Rhodococcus erythropolis*) [19–21]; Proteobacteria (*Agrobacterium*, *Ochrobactrum* sp., *Klebsiella pneumoniae*) [22, 23, 19] and Bacteroidetes) (*Chryseobacterium* sp., *Muricauda olearia*) [24, 25]. The AHL lactonases produced by all these bacteria belong to the metallo- β-lactamase, phosphotriesterase (PTE) and α/β hydrolase-fold families of proteins. The second group of QQ enzymes, the AHL acylases, catalyse hydrolytic cleavage of an amide bond in AHL to form homoserine lactone and free fatty acid. AHL acylases belong to the Ntn-hydrolase superfamily [26]. AHL acylases have been identified in Proteobacteria (*Vario*v*orax paradoxus*is, *Ralstonia* sp., *P*. *aeruginosa*, *Comamonas* sp.) [27–30] and Actinobacteia (*Streptomyces* sp.) [31].

Heterologous expression of bacterial QQ enzymes, either by pathogenic bacteria or by the host plant, has been shown to result in the attenuation of pathogen virulence. For instance, overexpression of *aiiA* from *Bacillus* sp. in *Erwinia carotovora* results in reduced AHL accumulation and significantly decreases secretion of the pectolytic enzymes which are a major contributor to infection [12]. Consistent with this observation, transforming *aiiA* into potato and tobacco plants results in enzymatic degradation of 3OC6HSL in the plant environment and reduced tissue maceration when plants were exposed to *E*. *carotovora* [32]. Finally, exposing plant to native bacteria that express QQ enzymes can prevent QS dependant infection to plant tissue by pathogens [33, 34]. This last strategy suggests that AHL degrading bacteria could be directly applied to plants as biocontrol agents against bacterial diseases.

In work carried out earlier in our laboratory AHL degrading bacteria were isolated from soil and plant roots to screen for effective biocontrol agent against soft rot causing Pcc. The soil isolate Gs50 identified here as *Lysinibacillus* sp. was selected for its ability to degrade synthetic C6HSL efficiently. The goal for the present study was to identify the mechanism of AHL degradation exhibited by the isolate Gs50 and to explore the QQ based biocontrol potential of isolate Gs50 for the attenuation of QS regulated pathogenesis in model of soft rot.

## Materials and Method

### Bacterial strains, media, growth conditions and chemicals

The soil isolate *Lysinibacillus* sp. Gs50 was grown in Luria-Bertani (LB) medium at 30°C. *Pectobacterium carotovorum* subsp. *carotovourm* BR1 (laboratory stock) was used as QS pathogen in the studies. It produces 3-oxo-hexanoyl homoserine lactone (3OC6HSL) which regulates the expression of the plant cell wall degrading enzymes polygalacturonase (PG) and pectin lyase (PNL); these enzymes cause soft rot in various host plants [35]. PccBR1 was grown in LB medium at 30°C. *Chromobacterium violaceum* CV026 was used as biosensor strain for detecting exogenous AHLs with acyl chains of C4 to C8 in length. *C*. *violaceum* CV026 produces purple pigment in response to short chain AHLs [36]. It was grown in LB medium with 30 μg ml^-1^ of Kanamycin. *Escherichia coli* strains DH5α and BL21 (DE3) were grown in LB medium at 37°C and 100μg ml^-1^ of Ampicillin was added when necessary. *N*-butanoyl-L-homoserine lactone (C4HSL), *N*-hexanoyl-DL-homoserine lactone (C6HSL), 3-oxo-hexanoyl-L-homoserine lactone (3OC6HSL), *N*-octanoyl-L-homoserine lactone (C8HSL) and 3-oxo-octanoyl-L-homoserine lactone (3OC8HSL) were purchased from Sigma-Aldrich.

### Identification of bacterial strain Gs50

Isolate Gs50 exhibiting the phenotype of AHL degradation was identified by 16S rRNA gene sequencing. The gene was amplified by PCR with following primers 27F (5'-AGAGTTTGATCCTGGCTCAG3') and 1541R (5'AAGGAGGTGATCCAGCCGCA3'). The amplicon was sequenced by Xcelris Genomics, India and analysed using NCBI BLAST.

### Bioassay for AHL degradation

The AHL degradation bioassay was performed according to Park et al., (2006) [37]. Briefly, overnight grown *Lysinibacillus* sp. Gs50 (~10^8^ CFU ml^-1^) culture was pelleted down and resuspended in phosphate buffer saline (PBS) pH 7.4. 20μl of *Lysinibacillus* sp. Gs50 cell suspension was added to 80μl of 25μM AHL (C4HSL, C6HSL, 3OC6HSL, C8HSL or 3OC8HSL in separate assays). This reaction mixture was incubated at 30°C for 2 hours under static conditions. Untreated controls of 25μM of C4HSL, C6HSL, 3OC6HSL, C8HSL and 3OC8HSL prepared in PBS were incubated at similar conditions. The amount of AHL remaining after exposure to *Lysinibacillus* sp. Gs50 was visualised by adding 30μl of reaction mixture supernatant to suspensions of the biosensor *C*. *violaceum* CV026 (0.002 OD corresponding to ~10^5^ CFU ml^-1^) in a 96 well microtiter plate. This was incubated overnight under static conditions at 30°C and the development of purple colour was observed.

To investigate whether AHL degrading enzyme is intracellular or extracellular, *Lysinibacillus* sp. Gs50 was grown overnight and culture supernatant was collected after centrifugation at 7000 rpm for 5 min. The culture pellet was resuspended in PBS. The resuspended cells were lysed using Sonics Vibracell TM sonicator (30% amplitude for 3 minutes at alternating intervals of 9 seconds) and supernatant and cell content were collected after centrifugation at 10,000 rpm for 10 min at 4°C. 20μl of culture supernatant, supernatant after sonication or cell pellet after sonication were assayed individually for AHL degradation by mixing with 80μl of 25μM C6HSL and incubating at 30°C for 2 hours under static conditions followed by the biosensor assay described above [25]. Reaction mixture containing whole cell of *Lysinibacillus* sp. Gs50 with 25μM of C6HSL was taken as degradation positive control and 25μM C6HSL was no treatment control.

An AHL restoration assay was performed as per Yates et al., (2002) [11]. For the assay, degradation reaction of overnight grown *Lysinibacillus* sp. Gs50 cells and 25μM of C6HSL was carried out for 2 hours at 30°C. Untreated control of 25μM of C6HSL was taken. After incubation, an aliquot of supernatant from both, reaction mixture and untreated control, was acidified with 25μl of 50mM HCl to bring the pH to 2. The reaction mixture and untreated control without acidification and after acidification were subjected to the biosensor assay.

The AHL degrading activity of appropriately induced *E*.*coli* BL21(DE3) pET22b(+)/*adeH* culture was assayed by 50μl of cell suspension with 50μl of 25 μM of C6HSL. Similar reactions were set up with *E*.*coli* BL21(DE3), *E*.*coli* BL21(DE3) pET22b(+) without IPTG induction, *E*.*coli* BL21(DE3) pET22b(+) with IPTG induction and *E*.*coli* BL21(DE3) pET22b(+)/*adeH* without IPTG induction as controls. 25μM of C6HSL was taken as no treatment control. Degradation of different chain length AHLs by appropriately induced *E*.*coli* BL21(DE3) pET22b(+)/*adeH* was carried out in a 100μl reaction mixture that contained 50μl of cell suspension and 50μl of 25μM of C4HSL, C6HSL, 3OC6HSL, C8HSL and 3OC8HSL. Untreated controls consisted of 25μM of C4HSL, C6HSL, 3OC6HSL, C8HSL and 3OC8HSL. The activity of purified AdeH was visualised by mixing 100μg of AdeH and 30μM of C6HSL in total volume of 150μl. Untreated control consisted of 30μM of C6HSL without purified AdeH. Biosensor assay was performed as above and appearance of purple pigment was observed. All bioassays for AHL degradation were conducted in triplicates.

### Growth on AHL as sole carbon source

To investigate whether *Lysinibacillus* sp. Gs50 was able to utilise AHL as sole carbon source, overnight grown *Lysinibacillus* sp. Gs50 culture was centrifuged at 7000 rpm and the pellet was washed three times with PBS and resuspended in 100μl of M9 minimal medium. This stock was used to inoculate (1% dilution) 1 ml aliquots of M9 minimal medium and supplemented with 2.5mM sucrose and 5mM C6HSL as carbon sources in the media [19]. *Lysinibacillus* sp. Gs50 was allowed to grow in shaking condition at 30°C. The optical density of the culture was monitored using Tecan InfinitePro 2000 microtiter-plate reader at 600nm till 50 hours. Uninoculated M9 was taken as negative control while M9 supplemented with 2.5mM was taken as positive control for the growth of *Lysinibacillus* sp. Gs50. The experiment was conducted in triplicates.

### Cloning and heterologous expression of the putative *adeH* gene

The hypothetical protein sequence identified by Kalia et al. (2011) for *Lysinibacillus sphaericus* C3-41 was taken as target to design primers. The *adeH* gene from the genomic DNA of *Lysinibacillus* sp. Gs50 was amplified by PCR with primers AdeH-F (5’: CG**GGATCC**GATGCGAATGGATAAGAATTA: 3’) and AdeH-R (5’: C**GAGCTC**GATTCATAATATCCTTCTGTCGATTT: 3’) (Sigma- Aldrich) using *pfu* polymerase. The sequences in bold show the restriction sites for *BamHI* in the forward primer and *SacI* in the reverse primer. PCR was carried out with initial denaturation at 94°C for 5 min followed 35 cycles of denaturation at 94°C for 50 sec, annealing at 47°C for 1 min and elongation at 72°C for 2 min with a final elongation at 72°C for 10 mins. The amplicon was incubated with *Taq* polymerase in the presence of dNTP’s at 72°C for 15 mins to include 3’-A overhangs, ligated using T4 DNA ligase into the pTZ57R/T cloning vector (Thermo Scientific) and transformed into *E*.*coli* DH5α cells. Clones were selected as white colonies on LA plates containing Ampicillin (100 μg/ml) and X- gal. For expression, pTZ57R/T/*adeH* was digested with *BamHI* and *SacI* and the purified DNA fragment ligated into the inducible expression vector pET22b(+) (Novagen) to give pET22b(+)/*adeH* which was transformed into *E*.*coli* BL21(DE3) cells. *E*.*coli* BL21(DE3) pET22b(+)/*adeH* culture was grown to an OD_600_ of 0.4, 0.5mM of IPTG (isopropyl-D-thiogalactopyranoside) was added to induce expression and cultures incubated at 30°C under shaking condition for further 6 hours. The expression of AdeH was detected by 10% SDS-PAGE.

### Purification of AHL degrading enzyme (AdeH)

His_6_-AdeH was purified to homogeneity using Ni-NTA affinity column chromatography. 100 ml induced *E*.*coli* BL21(DE3) pET22b (+)/*adeH* was centrifuged at 7000 rpm for 10 mins, resuspended in10 ml lysis buffer 1 (50mM Tris-Cl buffer pH 8.0, 1% sarcosine, 0.3% TEA, 0.5% triton X 100, 20mM imidazole and 300mM NaCl) and incubated on ice for 2 hours. Cells were sonicated (Sonics VibracellTM) at 30% amplitude for 10 minutes at alternating intervals of 9 seconds and then centrifuged at 7,000 rpm for 10 minutes at 4°C. The supernatant was added to a column containing Ni-NTA beads charged with lysis buffer 2 (50mM Tris-Cl buffer pH 8.0 and 300mM NaCl) and placed on rocker for 2 hours. The column was washed with 50 ml wash buffer (50mM Tris-Cl buffer pH 8.0, 20mM imidazole and 300mM NaCl). One ml elution buffer (50mM Tris-Cl buffer pH 8.0, 300mM imidazole and 300mM NaCl) was added and fractions collected after 15 min of incubation. The fractions were pooled and precipitated with acetone. Purified enzyme was resuspended in 50 mMTris-Cl buffer (pH 8.0) after acetone was evaporated and purity of the enzyme was assessed by 10% SDS-PAGE.

### Mass spectrometry analysis of the products of AHL degradation by AdeH

To determine the chemical structure of enzymatic reaction products of AdeH, C6HSL was subjected to degradation by purified AdeH and the resulting enzymatic reaction product was analysed by electrospray ionization (ESI)-mass spectrometry (MS). The reaction contained purified AdeH (~5μg) mixed with C6HSL (100μM) in 500μl of 50mM Tris-Cl buffer pH 8.0 [32, 38]. The no treatment control of only C6HSL (100μM) in 500μl of 50mM Tris-Cl buffer pH 8.0 was included. After 2 hours of incubation at 35°C, both the reaction mixture and no treatment control were extracted three times with 0.1% acidified ethyl acetate and the combined organic phase was evaporated in a rotary evaporator. The samples were dissolved in 100μl methanol, ionized by positive-ion electrospray and subjected to ESI-MS analysis in HCT Ultra PTM Discovery System (ETD II- Bruker Daltonics) with 1100 series HPLC (Agilent) at Indian Institute of Science, Bangalore, India.

### Characterization of enzyme activity of AdeH

To determine the substrate saturation curve, purified AdeH (2μg) was added to C6HSL (60, 120, 180, 240, 300, 600 picomol) in 50mM Tris-Cl buffer (pH 8.0) in a final volume of 6μl and incubated at 30°C for 2 hours. C6HSL (60, 120, 180, 240, 300, 600 picomol) in 50mM Tris-Cl buffer (pH 8.0) without purified AdeH were taken as no treatment controls. The effect of temperature on purified AdeH activity was determined by adding 2μg of AdeH to C6HSL (240 picomol) in a final volume of 6μl. Similarly C6HSL (240 picomol) in a final volume of 6μl without AdeH was no treatment control. Both, reaction mixtures and controls, were incubated at 5°C, 15°C, 20°C, 25°C, 30°C, 37°C, 45°C or 50°C in 50mM Tris-Cl buffer (pH 8.0). The effect of pH on purified AdeH activity was determined by adding 2μg of AdeH to C6HSL (240 picomol) in a final volume of 6μl. No treatment controls consisted of C6HSL (240 picomol) in a final volume of 6μl without purified AdeH. The reactions and controls were set up at different pH (3.6, 4.0, 4.6, 5.0, 5.6, 6.0, 6.6, 7.0, 7.6, 8.0, 8.6, 9.0) and were incubated 35°C. The thermal stability of purified AdeH was determined by pre-incubating the enzyme at different temperatures (10°C, 20°C, 30°C, 40°C and 50°C) for 2 hours, adding 240 picomol of C6HSL to the treated AdeH and incubating at 35°C for 2 hours. The effect of different metal ions (Cd2^+^, Mn2^+^, Fe2^+^, Zn2^+^, Mg2^+^, Cu2^+^, Ca2^+^) and EDTA, on the activity of purified AdeH was determined by adding 2μg of AdeH to C6HSL (240 picomol) and 1mM of different metal ions in a final volume of 6μl. A reaction of purified AdeH with 240 picomol of C6HSL without any cations or EDTA was set up as control. The reaction mixtures and controls were incubated at 35°C in 50mM Tris-Cl buffer (pH8.0). In each case, the reactions were stopped by adding 2% SDS and C6HSL was quantified by using the modified protocol for AHL quantification given by Zhang et al. (2007) [39]. The amount of remaining C6HSL was calculated by using the equation derived from the standard graph of bioassay (S1 Fig) and the amount of hydrolysed C6HSL was calculated. All experiments were carried out in triplicates. AdeH enzyme activity was defined as the hydrolysed picomols of C6HSL per minute.

### *In vitro* co-culture assay

*Lysinibacillus* sp. Gs50 (10^8^ CFU ml^-1^) and PccBR1 (10^8^ CFU ml^-1^) were co-inoculated in LB and incubated at 30°C under shaking conditions for 12 hours. PccBR1 was inoculated and grown alone as a control. The growth rate of PccBR1, its 3OC6HSL production, polygalacturonase and pectin lyase activity was monitored from the co-cultured and grown alone samples [35]. The CFU counts for *Lysinibacillus* sp. Gs50 and PccBR1 in the co-culture after 12 hours were recorded separately based on their distinct colony morphologies on Luria Agar (LA) plates. Similarly the CFU count was recorded for the PccBR1 grown alone culture. The supernatants from both, co-cultured and PccBR1 grown alone samples, were loaded into 8 mm wells cut into LA plates overlaid with *C*. *violaceum* CV026 and the amount of 3OC6HSL produced by PccBR1 was determined by the size of the purple coloured zone around the well. To assay polygalacturonase (PG) activity, 100μl of culture supernatant from PccBR1 alone and from the co-culture sample was incubated with 200μl 0.5% (w/v) polygalacturonic acid (in 50mM Tris-Cl buffer pH 8.0) at 40°C for 30 min. The amount of reducing sugar released by hydrolysis of polygalacturonic acid was measured at 540nm after adding dinitrosalicylic acid (DNS) reagent. One Unit of enzyme activity was defined as the amount of enzyme required to release 1μmole of D-galacturonic acid per min in optimum condition. To assay pectin lyase (PNL) activity, 100μl of culture supernatant from PccBR1 alone and from the co-culture sample was incubated with 250μl of 0.5% (w/v) pectin (in 50 mMTris-Cl buffer pH 8.0 and 5mM EDTA) at 40°C for 1 hour. Unsaturated oligosaccharides released as a result of the cleavage of polygalacturonic acid was measured after adding thiobarbituric acid (TBA) reagent at 550nm [40]. One unit of PNL activity was defined as the amount of enzyme required to change O.D_550_ by 0.01 in the absorbance per hour under the assay conditions. The co-culture experiment was carried out in three independent trials wherein each trial had three replicates for CFU count, polygalacturonase and pectin lyase activities.

### Soft rot attenuation assay

Potatoes, carrots and cucumbers were purchased from local market and washed thoroughly with running tap water. Surface sterilization was carried out by immersing the vegetables sequentially in 0.01% mercury chloride and 70% ethanol then rinsing them with sterile distilled water. The vegetables were cut into slices of 4–5 mm thickness and weighed [41]. *Lysinibacillus* sp. Gs50 (~1.12 ×10^8^ CFU ml^-1^) and PccBR1 (~2.8 ×10^6^ CFU ml^-1^), were mixed and 5μl of the mixture was injected into each slice. The slices were placed in sterile Petri dishes and incubated at 30°C for 24 hours. The area of macerated tissue (mm^2^) was calculated from the diameter of the macerated region. Macerated tissue was scooped out and weighed. Percentage maceration was calculated by comparing this with the initial weight of tissue at the start of the assay. As a positive control, the PccBR1 was inoculated alone and as a negative control (mock treated control) slices were inoculated with sterile PBS. The same assay was performed with appropriately induced *E*.*coli* BL21(DE3) pET22b(+)/*adeH* and PccBR1 on potato slices only. In this case, controls consisted of PccBR1 alone, IPTG induced *E*.*coli* BL21(DE3) pET22b(+) and PccBR1, mock inoculated with PBS. Three independent trials were carried out. Each trial had three replicates.

We performed preventive and curative biocontrol assays on potato slices, in two separate sets of experiments. *Lysinibacillus* sp. Gs50 was either added 12 hours before (preventive biocontrol) or 12 hours after (curative biocontrol) the pathogen PccBR1 was applied to the potato slices [42]. Controls consisted of inoculating PccBR1 alone and mock inoculated with PBS. Three independent trials were carried out and each trial consisted of three replicate potato slices in separate Petri plates.

### Statistical analysis

The data on the QQ effects of *Lysinibacillus* sp. Gs50 on virulence factors production and control of soft rot caused by PccBR1 were analysed using Two- tailed unpaired T tests in Graphpad Prism software (version 6). Differences at *p* < 0.05 were considered significant.

### Nucleotide sequence accession numbers

The sequences of the *adeH* and 16S rRNA gene of *Lysinibacillus* sp. Gs50 have been deposited in GenBank with accession numbers KU219945 and KR709144, respectively.

## Results

### Characterization of AHL degrading *Lysinibacillus* sp. Gs50

**Fig 1:**
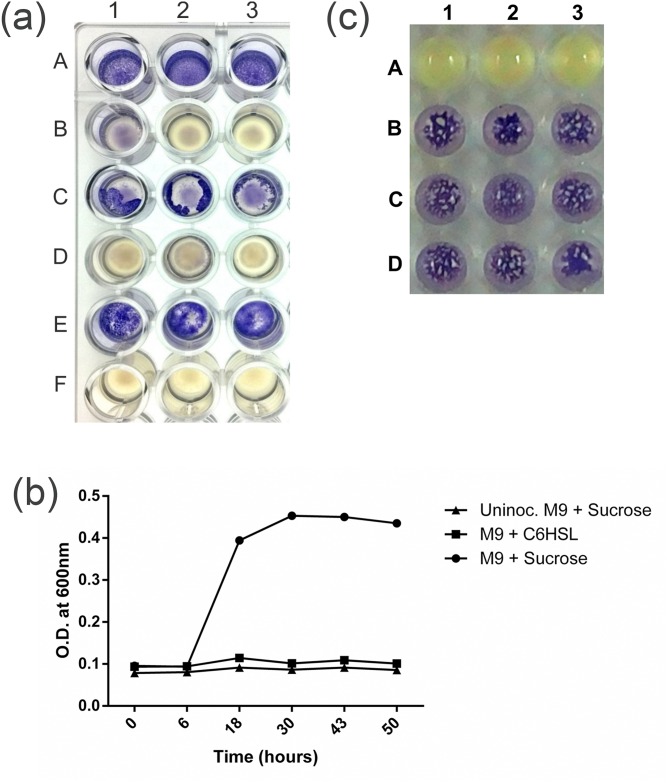
Characterization of AHL degrading *Lysinibacillus* sp. Gs50. **(a)** AHL degrading enzyme in *Lysinibacillus* sp. Gs50 cells. 25μM C6HSL was added to culture supernatant (A 1, 2, 3), sonicated pellet (B 1, 2, 3), supernatant after sonication (C 1, 2, 3) and whole cell of *Lysinibacillus* sp. Gs50 (D 1, 2, 3). *C*. *violaceum* CV026 (E 1, 2, 3) + 25μM C6HSL is no treatment control and only *C*. *violaceum* CV026 (F 1, 2, 3) is biosensor control **(b)** Growth of *Lysinibacillus* sp. Gs50 in minimal medium containing C6HSL as sole carbon source assessed based on OD_600_. Values are represented as mean ±SD for three replicates **(c)** Recovery of *Lysinibacillus* sp. Gs50 degraded C6HSL by acidification. Reaction mixture of *Lysinibacillus* sp. Gs50 + 25μM C6HSL (A 1, 2, 3) and acidified reaction mixture of *Lysinibacillus* sp. Gs50 + 25μM C6HSL with 25μl of 50mM HCl (B 1, 2, 3). *C*. *violaceum* CV026 + 25μM C6HSL (C 1, 2, 3) and acidified *C*. *violaceum* CV026 + 25μM C6HSL (D 1, 2, 3) are controls.

Among 97 soil and root associated bacterial isolates screened for their ability to degrade AHL, isolate Gs50 was selected for further studies as it was able to completely inactivate the C6HSL in 2 hours. In present study, partial sequence of 16S rRNA gene of the isolate Gs50 was found to be 99% similar to those of *Lysinibacillus macrolides*, *Lysinibacillus boronitolerance* and *Lysinibacillus fusiformis* therefore it was designated *Lysinibacillus* sp. Gs50. Further AHL degradation bioassay performed with different AHLs as substrates suggested that *Lysinibacillus* sp. Gs50 has the ability to inactivate different AHLs (S2 Fig). In order to find out if the AHL degrading enzyme from *Lysinibacillus* sp. Gs50 was intracellular or extracellular, the activity was observed in different cellular fractions which are Culture supernatant, Supernatant after sonication and Pellet after sonication. *C*. *violaceum* CV026 failed to develop purple colour in the wells containing reaction mixture of pellet after sonication. This indicated that the AHL degrading activity was associated with the pellet after sonication fraction (Fig 1a). While *C*. *violaceum* CV026 developed purple colour due to the presence of intact C6HSL in case of culture supernatant and the supernatant after sonication fractions indicating absence of AHL degrading activity (Fig 1a). Based on this, it can be inferred that the AHL degrading enzyme in *Lysinibacillus* sp. Gs50 was not extracellular but was intracellular. The results suggest that even though the AHL degrading enzyme was intracellular there is a strong likelihood of it being associated with the lysed cell pellet and not the cytosol. If the AHL is cleaved by amidohydrolysis, the resulting products can serve as carbon source for the growth of the bacteria. However, *Lysinibacillus* sp. Gs50 could not utilise C6HSL provided in minimal medium as sole carbon source as it demonstrated no growth on C6HSL whereas it showed significant growth when sucrose was provided as sole carbon source (Fig 1b). If the cleavage of AHL is due to enzymatic/non-enzymatic lactone ring hydrolysis, it can be restored to intact AHL at acidic pH. As observed, C6HSL degraded by *Lysinibacillus* sp. Gs50 showed reappearance of purple colour upon acidification (Fig 1c). This suggested that *Lysinibacillus* sp. Gs50 probably cleaved the lactone ring of AHL which was restored to intact at low pH. Summing up from the results obtained, it was concluded that AHL degradation activity of *Lysinibacillus* sp. Gs50 may be due to intracellular AHL lactonase.

### Identification of gene responsible for AHL degradation in *Lysinibacillus* sp. Gs50

**Fig 2:**
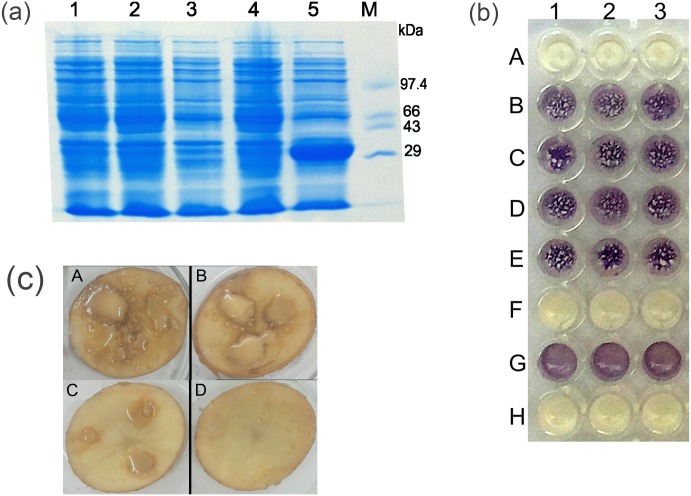
Characterization of *E*.*coli* BL21(DE3) pET22b(+)/*adeH*. **(a)** SDS PAGE of *E*.*coli* BL21(DE3) pET22b(+) expressing AdeH. Lane 1: *E*.*coli* BL21(DE3) lysate, Lane 2: Lysate of *E*.*coli* BL21(DE3) pET22b(+) without IPTG induction, Lane 3: Lysate of *E*.*coli* BL21(DE3) pET22b(+) with IPTG induction, Lane 4: Lysate of *E*.*coli* BL21(DE3) pET22b(+)/*adeH* without IPTG induction, Lane 5: Lysate of *E*.*coli* BL21(DE3) pET22b(+)/*adeH* with IPTG induction, M: Protein molecular weight marker. **(b)** AHL degradation assay of *E*.*coli* BL21(DE3) pET22b(+)/*adeH*. 25μM C6HSL was added to *Lysinibacillus* sp. Gs50 (A 1, 2, 3), *E*.*coli* BL21(DE3) (B 1, 2, 3), *E*.*coli* BL21(DE3) pET22b(+) without IPTG induction (C 1, 2, 3), *E*.*coli* BL21(DE3) pET22b(+) with IPTG induction (D 1, 2, 3), *E*.*coli* BL21(DE3) pET22b(+)/*adeH* without IPTG induction (E 1, 2, 3), *E*.*coli* BL21 (DE3) pET22b(+)/*adeH* with IPTG induction (F 1, 2, 3). *C*. *violaceum* CV026 (G 1, 2, 3) + 25μM C6HSL is no treatment control and only *C*. *violaceum* CV026 (H 1, 2, 3) is biosensor control. **(c)** Soft rot attenuation assay of *E*.*coli* BL21 (DE3) pET22b(+)/*adeH* caused by PccBR1. Potato slices were inoculated with A: PccBR1, B: IPTG induced *E*.*coli* BL21(DE3) pET22b(+) and PccBR1, C: IPTG induced *E*.*coli* BL21 (DE3) pET22b(+)/*adeH* and PccBR1, D: PBS control.

Kalia et al. (2011) mined sequenced whole genome database and revealed a list of organisms possessing conserved domains of AHL lactonases [43]. Accordingly, *Lysinibacillus sphaericus* C3-41 genome showed the conserved domain of AHL lactonase. We identified a sequence of hypothetical protein encoded by bsph_3377 in *Lysinibacillus sphaericus* C3-41 (NCBI Reference Sequence: NC_010382.1) genome showing presence of the conserved domain of AHL lactonase. As the isolate Gs50 belonged to *Lysinibacillus* genus, primers were designed to target the gene sequence of hypothetical protein encoded by bsph_3377 and used for amplification of the AHL lactonase gene from the genomic DNA of *Lysinibacillus* sp. Gs50. The expected size 792 bp fragment was amplified and cloned in pTZ57R/T, subcloned in pET22b(+) followed by transformation in *E*.*coli* BL21(DE3) (designated *E*.*coli* Bl21(DE3) pET22b(+)/*adeH*) for its expression. Upon IPTG induction of *E*.*coli* Bl21(DE3) pET22b(+)/*adeH*, a distinct band of ~29 kDa protein was observed on SDS PAGE gel (Fig 2a) and other controls viz. *E*.*coli* BL21(DE3), *E*.*coli* BL21(DE3) pET22b(+) without IPTG induction, *E*.*coli* BL21(DE3) pET22b(+) with IPTG induction and *E*.*coli* BL21(DE3) pET22b(+)/*adeH* without IPTG induction did not show the protein band of interest. C6HSL degrading activity shown by *E*.*coli* Bl21(DE3) pET22b(+)/*adeH* was comparable to the activity of wild type *Lysinibacillus* sp. Gs50 (Fig 2b) indicating that the cloned gene had imparted AHL degradation phenotype and it was therefore designated *adeH* (AHL degrading hydrolase). Moreover, *E*.*coli* BL21(DE3) pET22b(+)/*adeH* was able to degrade different chain length AHLs, like the wild type *Lysinibacillus* sp. Gs50 (S2 Fig). Further, *E*.*coli* Bl21 (DE3) pET22b(+)/*adeH* when co-inoculated with PccBR1 on the potato slice resulted in the virulence attenuation of PccBR1 as it decreased the soft rot symptoms caused by PccBR1 (Fig 2c). These results confirmed that the cloned gene from *Lysinibacillus* sp. Gs50 was the one which encoded the AHL degrading enzyme.

### AdeH is an AHL lactonase

**Fig 3:**
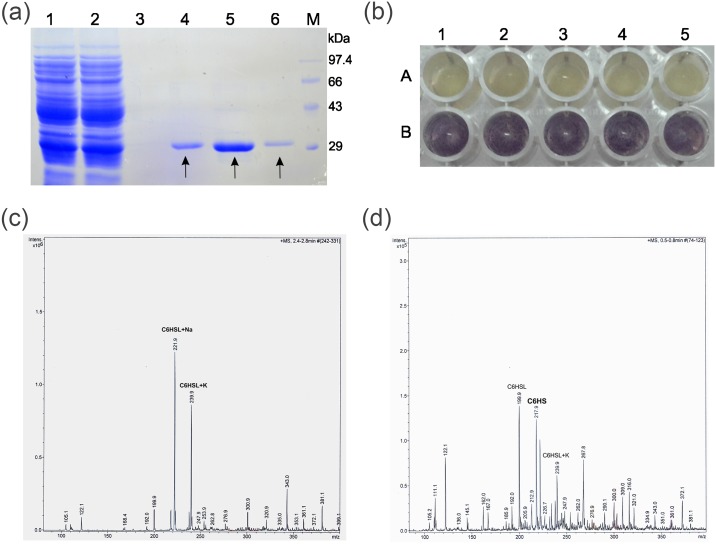
Mechanism of AdeH catalysis. **(a)** SDS PAGE analysis of purified His_6_ tagged AdeH. Lane 1: Unbound Fraction, Lane 2: First wash fraction, Lane 3: Last wash fraction, Lane 4: First elution fraction (AdeH), Lane 5: Second elution fraction (AdeH), Lane 6: Third elution fraction (AdeH), M: Protein molecular weight marker **(b)** AHL degradation assay of purified AdeH. 30 μM C6HSL treated with 100 μg of purified and acetone precipitated AdeH (A 1, 2, 3, 4, 5) and no treatment control of 30 μM C6HSL (B 1, 2, 3, 4, 5) were exposed to *C*. *violaceum* CV026 **(c)** ESI-MS analysis of C6HSL **(d)** ESI-MS analysis of C6HSL degraded by AdeH showing a peak of C6HS.

Purification of AdeH from *E*.*coli* BL21(DE3) pET22b(+)/*adeH* was carried out using Ni affinity chromatography as the AdeH expressed in pET22b(+) had a 6-His tag at its C terminal. The purified AdeH was detected on 10% SDS PAGE. The purified protein resulted in a single band of ~29 kDa molecular weight (Fig 3a). Further the purified and acetone precipitated AdeH completely degraded the C6HSL which confirmed that purified AdeH was the enzyme responsible for AHL degradation (Fig 3b). To elucidate the mechanism of AdeH catalysis, C6HSL was treated with purified AdeH and the resulting degradation products were analysed. The ESI-MS profile of C6HSL (substrate for enzymatic reaction) showed peaks of Na- conjugate and K- conjugates of C6HSL having an m/z of 221.9 (M + Na) and 239.9 (M + K) respectively (Fig 3c). Subtraction of the mass of conjugates from their m/z values yielded the m/z of 199.9 which corresponds to that of C6HSL. The enzymatic product of AdeH reaction with C6HSL as substrate gave a peak with m/z of 217.9 showing a mass increase of 18 as compared to the m/z of intact C6HSL (199.9). The m/z 217.9 represented the *N*- hexanoyl homoserine (C6HS) which is obtained by the addition of a water molecule to the ester bond of *N*- hexanoyl homoserine lactone (C6HSL) as a result of the hydrolytic cleavage of lactone ring (Fig 3d). This result confirmed that AdeH hydrolyses the lactone ring of C6HSL and hence proved to be an AHL lactonase.

**Fig 4:**
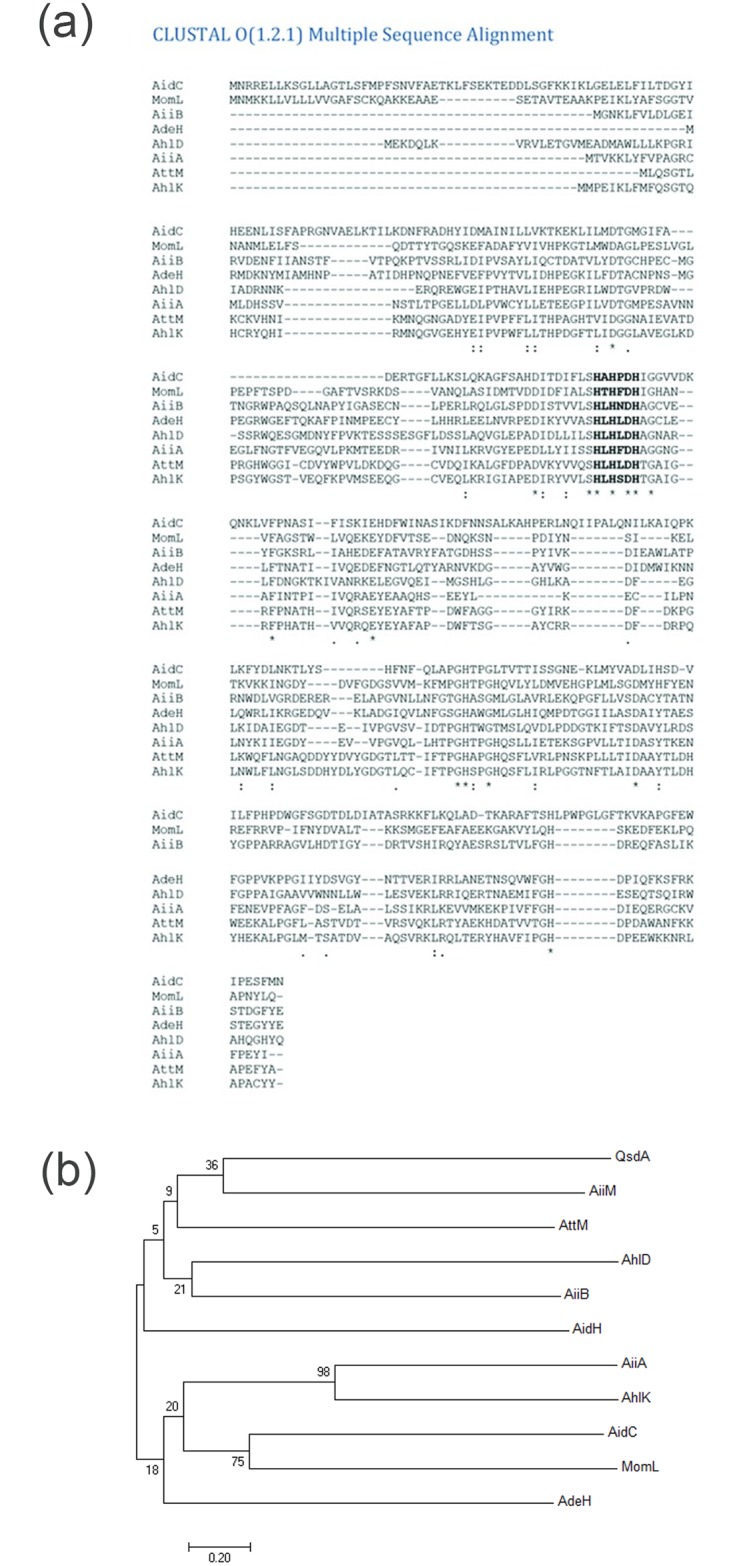
Relationship of AdeH with other AHL lactonases. **(a)** Multiple sequence alignment of AdeH and other representative AHL lactonases. Sequence alignment was performed by Clustal Omega online software. Representative AHL lactonases are AidC (Accession: BAM28988), MomL (Accession: AIY30473), AiiB (Accession: NP_396590), AhlD (Accession: AAP57766.1), AiiA (Accession: AAF62398.1), AttM (Accession: AAD43990.1) and AhlK (Accession: AAO47340.1). The shared “HXHXDH” motifs highlighted in bold. Asterisk indicates positions which have a single, fully conserved residue. Colon indicates conservation between groups of strongly similar properties. Period indicates conservation between groups of weakly similar properties. **(b)** Neighbor-joining tree of AHL lactonases belonging to the metallo- β-lactamase, phosphotriesterase and α/β hydrolase-fold family based on amino acid sequences. The dendrogram was constructed by neighbor joining method with the ClustalW program in the MEGA 7 software package (1,000 bootstrap replicates). Scale bar, 0.2 substitutions per amino acid position.

Sequencing of the purified AdeH provided the amino acid sequence which was similar to the sequence of the translated 264 amino acid protein sequence obtained in *in silico* analysis of the targeted *adeH* gene. The deduced amino acid sequence of AdeH was used to perform local BLASTP search in NCBI database which revealed that it showed 99% identity with MBL fold metallo-hydrolase from strains *Lysinibacillus* sp. F5 (accession number WP 058844592.1), *Lysinibacillus sphaericus* (accession number WP 054550471.1) and *Lysinibcillus fusiformis* (accession number WP 004231440.1) which suggested that AdeH belongs to the metallo- β-lactamase superfamily of protein. Many known AHL lactonases belong to this superfamily of proteins. The amino acid sequence of AdeH was compared with those of the known AHL lactonases of metallo- β-lactamase superfamily through multiple sequence alignment. The zinc-binding motif “HXHXDH” which is conserved among them was also found in the amino acid sequence of AdeH (Fig 4a). To determine the relationship between AdeH and the known representative AHL lactonases from various bacteria, the amino acid sequence of AdeH was subjected to the phylogenetic analysis using the neighbor-joining method in Mega7 software. The representative amino acid sequences taken were AiiA from *Bacillus* sp. 240B1, AttM and AiiB from *A*. *tumefaciens* C58, AhlD from *Arthrobacter* sp. IBN110, AhlK from *Klebsiella pneumoniae*, AidC from *Chryseobacterium* sp. StRB126 and MomL from *Muricauda olearia* belonging to metallo- β-lactamase superfamily; QsdA from *R*. *erythropolis* W2 which belong to phosphotriesterase (PTE) family; AiiM from *Microbacterium testaceum* StLB037 and AidH from *Ochrobactrum* sp. T63 belonging to the α/β hydrolase-fold family. Although AdeH belongs to the metallo- β-lactamase superfamily it was distant from the other AHL lactonase clusters representatives of metallo- β-lactamase superfamily (Fig 4b).

### Biochemical characterization of AdeH

**Fig 5:**
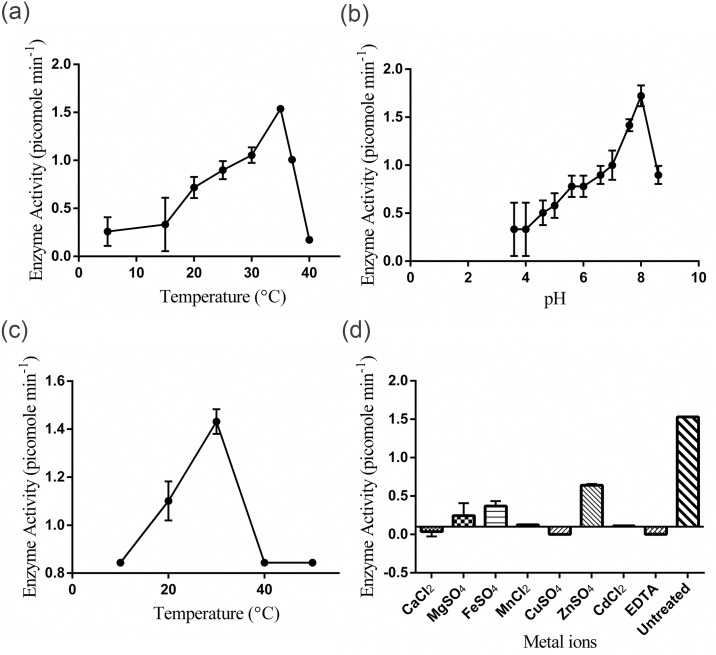
Biochemical characterization of AdeH. **(a)** Effect of Temperature on AdeH activity **(b)** Effect of pH on AdeH activity **(c)** Thermal stability of AdeH **(d)** Effect of cations and EDTA on AdeH activity. Values represent the mean of three replications. Bars indicate standard deviation of the mean.

The effect of physical and chemical parameters such as pH, temperature, EDTA and metal ions that may affect the enzyme activity of purified AdeH was investigated. AdeH could exhibit C6HSL degrading activity in the temperature range of 20°C to 37°C. The optimum temperature for AdeH activity was 35°C where the maximum activity of 1.5 picomol min^-1^ was achieved. Thereafter the activity declined at temperature 40°C because of enzyme inactivation (Fig 5a). AdeH could exhibit C6HSL degrading activity in the pH range from 5 to 8.6 and the activity was enhanced with increase in pH from 5 to 8. The AdeH activity reached maximum at pH 8 giving 1.8 picomol min^-1^ activity (Fig 5b). The activity declined slightly as the pH was raised to 8.6 and at pH 9 AdeH activity was completely abolished. The potential interference of non-enzymatic pH-dependent lactone hydrolysis was precluded by analysis of the controls in which same amount of C6HSL was incubated in the reaction buffers of different pH without the purified AdeH (S3 Fig). AdeH appeared unstable at pH 9, hence it failed to degrade C6HSL. Moreover, little activity was detected when pH was adjusted below 5. Purified AdeH exhibited excellent thermal stability at 30°C as it maintained maximum activity of 1.4 picomol min^-1^. There was a reduction of 50% activity when subjected to 20°C for 2 hours. AdeH was not stable at low and high temperatures as it did not maintain any activity at 10°C and 40°C and above (Fig 5c). Various metal ions Ca^2+^, Mn^2+^, Cu^2+^ and Cd^2+^ completely inhibited AdeH at 1mM concentration and AdeH activity was abolished in the presence of these cations. On the other hand, Mg^2+^, Fe^2+^ and Zn^2+^ partially inhibited AdeH activity and decreased it to different extents at 1mM concentration compared to AdeH activity without any metal ion which was found out to be maximum of 1.5 picomol min^-1^ (Fig 5d). Chelating agent EDTA at 1mM concentration completely abolished the AdeH activity (Fig 5d). AHL degradation kinetics of AdeH was determined by plotting velocity versus substrate concentration (S3 Fig). The *K*_*M*_ was calculated by fitting the data to the Michaelis-Menten equation. AdeH showed *K*_*M*_ value of 3.089 μM for C6HSL at pH 8.0 and 35°C while the specific activity was 0.8 picomol min^-1^μg^-1^.

### Quorum quenching potential of *Lysinibacillus* sp. Gs50 in co-culture with PccBR1

**Fig 6:**
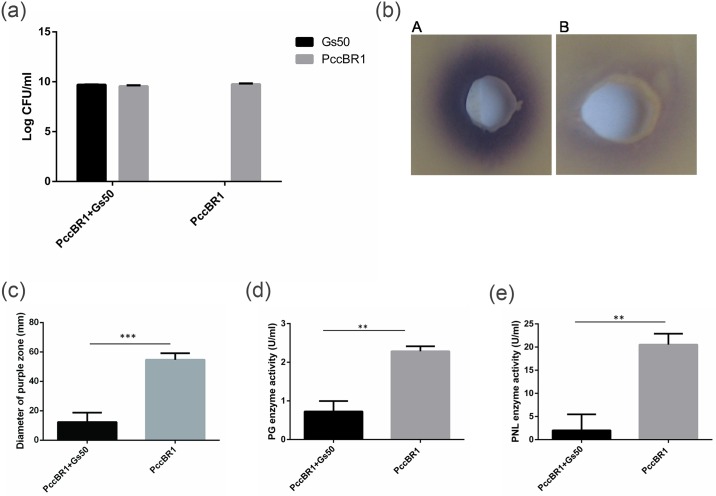
*In vitro* co-culture of *Lysinibacillus* sp. Gs50 and PccBR1. **(a)** Influence of *Lysinibacillus* sp. Gs50 on the growth of PccBR1 in co-culture. **(b)** 3OC6HSL production by PccBR1 in the co-culture with *Lysinibacillus* sp. Gs50. Presence of 3OC6HSL is indicated by purple zone produced by biosensor *C*. *violaceum* CV026 around the well **(c)** Diameter of the purple zone in mm from the supernatant of *Lysinibacillus* sp. Gs50 and PccBR1 co-culture and PccBR1 alone **(d)** Polygalacturonase (PG) activity in the supernatant of *Lysinibacillus* sp. Gs50 and PccBR1 co-culture **(e)** Pectin lyase (PNL) activity in the supernatant of *Lysinibacillus* sp. Gs50 and PccBR1 co-culture. Values represent the mean of three replications. Bars indicate standard deviation of the mean. Statistical analysis was done using two-way unpaired t- test (** = p < 0.01).

To investigate whether *Lysinibacillus* sp. Gs50 was able to reduce the virulence factors controlled by QS in Pcc, co-culture of *Lysinibacillus* sp. Gs50 and PccBR1 at similar CFU ml^-1^ was carried out. After 12 hours it was observed that the growth of PccBR1 in co-culture was same as PccBR1 grown alone (~10^9^ CFU ml^-1^) demonstrating that the growth of PccBR1 was not influenced by *Lysinibacillus* sp. Gs50 (Fig 6a) while the 3OC6HSL produced by PccBR1when co-cultured with *Lysinibacillus* sp. Gs50 was decreased compared to PccBR1 grown alone. This is depicted in Fig 6b and 6c where the purple zone produced by the culture supernatant of co-culture is smaller as compared to the purple zone produced by culture supernatant from PccBR1 alone. Similarly the activities of virulence determinant enzymes PG (polygalaturonase) and PNL (pectin lyase) of PccBR1supernatant were 2 U ml^-1^ and 20 U ml^-1^ respectively which decreased significantly to 0.6 U ml^-1^ and 3 U ml^-1^ in the supernatant of the co-culture sample (Fig 6d and 6e). It could be concluded that growth of PccBR1 was not affected in the presence of *Lysinibacillus* sp. Gs50. Further, AHL lactonase (AdeH) produced by *Lysinibacillus* sp. Gs50 degraded the QS molecule 3OC6HSL produced by PccBR1 and resulted in decreased PG and PNL activities, the production of which is regulated by 3OC6HSL in PccBR1.

### Biocontrol potential of AHL lactonase producing *Lysinibacillus* sp. Gs50

**Fig 7:**
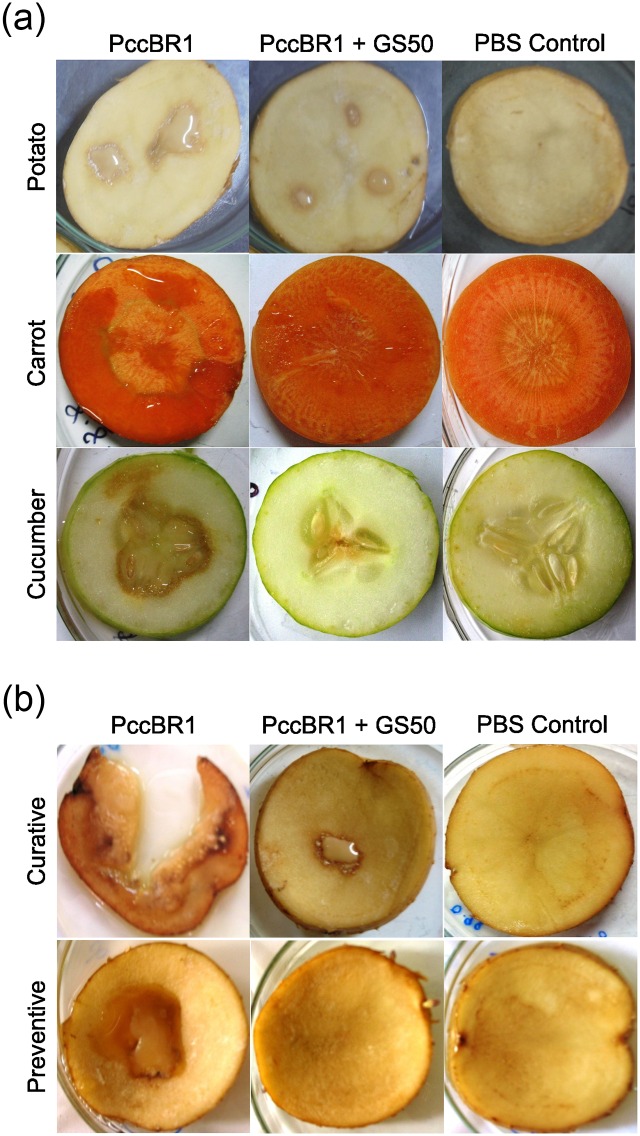
Quorum quenching based biocontrol potential of *Lysinibacillus* sp. Gs50 against soft rot causing PccBR1. **(a)** Soft rot attenuation assay on different hosts (Potato, Carrot, Cucumber) of PccBR1, co-inoculated with *Lysinibacillus* sp. Gs50 and PccBR1. PccBR1 alone is pathogen control and PBS is no infection control **(b)** Curative and preventive quorum quenching based biocontrol potential of *Lysinibacillus* sp. Gs50. In curative soft rot attenuation assay the pathogen PccBR1 was inoculated 12 hours before *Lysinibacillus* sp. Gs50 on potato slices. In preventive soft rot attenuation assay *Lysinibacillus* sp. Gs50 was inoculated 12 hours before the pathogen PccBR1. PBS inoculated slices were taken as no infection control.

The QQ based biocontrol potential of AHL lactonase producing *Lysinibacillus* sp. Gs50 on different hosts of soft rot causing PccBR1 was evaluated. Simultaneous inoculation of *Lysinibacillus* sp. Gs50 and PccBR1on potato resulted in 3 and 4 fold decrease in maceration area and macerated tissue (%) respectively compared to the maceration caused by PccBR1 alone (Fig 7a, Table 1). Carrot slices inoculated with *Lysinibacillus* sp. Gs50 and PccBR1 showed 5 and 7 fold reduction in maceration area and macerated tissue (%) respectively than the maceration caused by only PccBR1 (Fig 7a, Table 1). The maceration on cucumber slices which were inoculated with *Lysinibacillus* sp. Gs50 and PccBR1 resulted in 6 fold decrease in both maceration area and macerated tissue (%) compared to PccBR1 alone (Fig 7a, Table 1). The reduction in maceration was significant in the case of both carrot and cucumber. Moreover, for curative biocontrol the potato slices were inoculated with *Lysinibacillus* sp. Gs50 12 hours after the inoculation of PccBR1. The AHL lactonase producing *Lysinibacillus* sp. Gs50 was able to restrict the further spread of the infection which is reflected in terms of 4 and 7 fold decrease in maceration area and macerated tissue (%) respectively compared to PccBR1 alone (Fig 7b, Table 1). For preventive biocontrol PccBR1 was inoculated 12 hours after *Lysinibacillus* sp. Gs50 which resulted in no maceration of potato slices (Fig 7b, Table 1). These studies highlight the potential of AHL lactonase producing *Lysinibacillus* sp. Gs50 as a biocontrol agent which can be applied to a broad host range susceptible to the QS pathogen PccBR1 and demonstrates the biocontrol ability of *Lysinibacillus* sp. Gs50 pre and post infection.

**Table 1:** Quorum quenching based biocontrol potential of *Lysinibacillus* sp. Gs50 against soft rot causing PccBR1.

	PccBR1		PccBR1 + *Lysinibacillus* sp. Gs50	
	Maceration Area (mm^2^)^a^	Macerated Tissue (%)^a^	Maceration Area (mm^2^)^a^	Macerated Tissue (%)^a^
Potato	26.28 ± 4.191	6.948 ± 1.817	7.244 ± 0.2326*	2.614 ± 0.3543
Carrot	70.62 ± 9.442	10.57 ± 0.6720	10.95 ± 3.078*	2.404 ± 0.1021**
Cucumber	457.3 ± 29.92	24.00 ± 2.070	72.86 ± 51.89**	3.608 ± 0.9988**
Curative	163.3 ± 13.17	42.63 ± 5.148	41.19 ± 19.28**	6.117 ± 1.334**
Preventive	32.66 ± 2.634	8.526 ± 1.029	No maceration	No maceration

^**a**^ Quantification of tissue maceration in terms of maceration area (mm^2^) and macerated tissue (%). Values represent the mean ± SEM of three independent trials. Each trial has three replicates. Statistical analysis was done using two-way unpaired t-test.

* depicts significant reduction in maceration area and macerated tissue (%) by *Lysinibacillus* sp. Gs50 treatment compared to PccBR1 alone treatment (* = *p*< 0.05, ** = *p* < 0.01).

## Discussion

Soil isolate Gs50 with remarkable AHL degrading activity, selected after screening of 97 isolates in earlier work, was identified as *Lysinibacillus* sp. *Lysinibacillus* is a Gram-positive, rod-shaped, and round-spore forming genus belonging to Firmicutes phylum. Organisms in this genus were previously regarded as members of the genus *Bacillus*, but the taxonomic status of these microorganisms was changed to the genus *Lysinibacillus* in 2007 [44]. This is the first report of AHL degrading ability from genus *Lysinibacillus* that has included the characterisation and subsequent identification of the enzyme responsible for it. Major QQ enzymatic mechanisms operate either through lactone hydrolysis carried out by AHL lactonases or amidohydrolysis carried out by AHL acylases. AHL lactonases hydrolyse the lactone ring of AHL, yielding the corresponding *N*-acyl homoserine which can be restored to *N*-acyl homoserine lactone at acidic pH [11]. In accordance with this, the product of C6HSL degradation after treatment with *Lysinibacillus* sp. Gs50 could be restored at pH 2 after addition of HCl. This suggested the possibility of *Lysinibacillus* sp. Gs50 producing with broad specificity and the AHL degrading enzyme was found located intracellularly. Interestingly, the activity was not detected in the cytosol but was associated with the pellet after sonication. Majority of the AHL lactonases are either cytoplasmic or intracellular [45] and display a wide spectrum for the substrate by cleaving different carbon acyl side chain length AHLs [2, 38, 46, 47]. AHL degradation products have been shown to serve as a source of nitrogen or/and carbon. *V*. *paradoxus* and AHL lactonase (AhlD) producing *Arthrobacter* sp. strains utilize the fatty acid released from AHL as an energy source [19, 27]. *Lysinibacillus* sp. Gs50 could not utilize C6HSL as carbon source and was unable to grow on it.

The AHL degradation mechanism of *Lysinibacillus* sp. Gs50 was identified by cloning a gene encoding for AHL lactonase (*adeH*) and its heterologous expression in *E*.*coli* BL21 (DE3). *E*.*coli* Bl21 (DE3) pET22b(+)/*adeH* also showed broad specificity like *Lysinibacillus* sp. Gs50. Moreover, it could decrease maceration symptoms appreciably when co-inoculated with PccBR1on potato slices. These observations demonstrated that the gene which was cloned in pET22b(+) imparted an AHL degradation phenotype to *E*.*coli* Bl21 (DE3) upon IPTG induction and in turn resulted in soft rot attenuation on potato slices. Further confirmation of AdeH as a lactonase was afforded by Mass spectrometry analysis of the AdeH enzymatic reaction products. AHL lactonases hydrolyse the lactone ring by the addition of water molecule into AHLs to produce acylhomoserine [18, 23, 32, 46, 48, 49]. Two peaks of Na and K substrate conjugates were visible in mass spectrum of C6HSL (substrate for AdeH) (Fig 3c). While the mass spectrum of AdeH reaction product gave a definite peak corresponding to acylhomoserine at 217.9 m/z in ESI-MS analysis clearly confirmed that AdeH catalysed lactone ring hydrolysis. Along with the product peak there were peaks of the substrate (C6HSL) at m/z 199.9 and of substrate conjugate (M + K) at m/z 239.9 (Fig 3d). These peaks might be the result of either incomplete reaction by AdeH or the possible recircularization of the hydrolysed product due to the presence of H^+^ during its run in positive mode of ionization. While MS analysis established that AdeH was AHL lactonase, *in silico* analysis revealed the presence of zinc-binding motif “HXHXDH” in the amino acid sequence of AdeH, which is conserved among AHL lactonases. Moreover, identity of AdeH to the known AHL lactonases from the metallo- β-lactamase superfamily were AiiA (30% identity) from *Bacillus* sp. 240B1, AttM (31% identity) and AiiB (40% identity) from *A*. *tumefaciens* C58, AhlD (30% identity) from *Arthrobacter* sp. IBN110, AhlK (31%) from *Klebsiella pneumoniae*, AidC (19%) from *Chryseobacterium* sp. StRB126 and MomL (24%) from *Muricauda olearia*. AdeH showed 26% identity to QsdA from *R*. *erythropolis* W2 which belong to phosphotriesterase (PTE) family. AdeH showed 30% identity to AiiM from *Microbacterium testaceum* StLB037 and 25% identity to AidH from *Ochrobactrum* sp. T63, the AHL lactonases belonging to the α/β hydrolase-fold family. QQ bacteria belonging to Firmicutes phylum such as *B*. *thuringiensis*, *B*. *cereus*, *B*. *anthracis*, and *B*. *mycoides*, *B*. *subtilis*, *B*. *amyloliquefaciens*, *Geobacillus* sp. [13–18] are reported to produce AiiA type of AHL lactonases. Surprisingly AHL lactonase (AdeH) of *Lysinibacillus* sp. Gs50 displayed only 30% identity with AiiA. Based on above mentioned identities and the phylogenetic relationship of AdeH with other AHL lactonase of metallo- β-lactamase, phosphotriesterase and α/β hydrolase-fold family, AdeH can be classified as distinctly different kind of AHL lactonase from metallo- β-lactamase family. Biochemical characterization of AdeH revealed its optimum temperature and range at 35°C and 10°C to 40°C respectively. The pH optimum and range was 8.0 and 3.6 to 8.6 respectively. Metal ions Mg^2+^, Ca^2+^, Mn^2+^, Fe^2+^, Cu^2+^, Cd^2+^, Zn^2+^ and EDTA had a negative effect on the enzyme activity at 1mM concentration. The *K*_*M*_ for C6HSL was 3.089 μM while the specific activity was 0.8 picomol of C6HSL degraded per minute per μg of AdeH.

In natural ecosystems a large diversity of organisms exists which produce enzymes for AHL degradation [50, 51] but a few natural isolates are studied to be used as biocontrol agents for reduction of QS regulated pathogenesis of bacterial infection [12,51, 52]. Therefore, the final goal of the study was to explore the potential use of AHL lactonase producing *Lysinibacillus* sp. Gs50 as biocontrol agent to attenuate QS regulated pathogenesis of PccBR1. The QS molecule of PccBR1 is 3OC6HSL and *Lysinibacillus* sp. Gs50 exhibited efficient 3OC6HSL degrading activity in AHL degradation assay therefore *in vitro* quorum quenching experiment was set up with *Lysinibacillus* sp. Gs50 and PccBR1. The results of this experiment established that *Lysinibacillus* sp. Gs50 was able to disrupt the QS regulated function in PccBR1 as was reflected by reduction in 3OC6HSL in co-culture compared to PccBR1 alone which in turn caused reduction in virulence enzyme (polygalacturonase and pectin lyase) activities of PccBR1. It was verified that this phenomena was not due to growth inhibition of PccBR1, as PccBR1 exhibited similar growth in co-culture with *Lysinibacillus* sp. Gs50 as was in PccBR1 alone. PccBR1 has a broad host range and can infect plants belonging to several different families, e.g. Apiaceae (carrot), Solanaceae (potato, eggplant) and Cucurbitaceae (cucumber, sankeguard). *Lysinibacillus* sp. Gs50 when co-inoculated with PccBR1 on potato, carrot and cucumber in maceration assays showed significant attenuation of soft rot symptoms in all of the hosts, implying that it has potential as broad-range biocontrol agent exhibiting QQ. Further, the ability to control symptoms pre and post infection is a valuable trait in a biocontrol agent, and *Lysinibacillus* sp. Gs50 demonstrated both successful preventive and curative biocontrol of PccBR1 on potato slices. In AHL degradation assays *Lysinibacillus* sp. Gs50 showed degradation of varying chain length AHLs with different efficiencies which suggests that AHL lactonase producing *Lysinibacillus* sp. Gs50 may interfere with QS regulated functions of other bacteria which may not be pathogenic if the isolate is applied as biocontrol agent in the rhizosphere of host plant. This kind of non-target effect of QQ based biocontrol approach on the beneficial traits of QS bacteria has been demonstrated by Molina et al., (2003) [42] and suggested by Cirou et al., (2007) [52]. The QS signalling molecules diffuse out to the extracellular environment for communication with other bacteria and the diffusion range of AHLs in the tomato rhizosphere is measured up to ~78μm [53] and the AHL lactonase produced by *Lysinibacillus* sp. Gs50 is intracellular. In this scenario the spatial structure and vicinity of the QQ bacteria (*Lysinibacillus* sp. Gs50) and the QS bacteria (pathogenic/beneficial) in the rhizosphere of host plant will play major role to achieve the balanced outcome of QQ based biocontrol approach. Hence the appropriate use of QQ biocontrol agents to get optimum and desirable effect needs further evaluation. The present study emphasises the utility of natural soil isolate as biocontrol agent with efficient AHL degrading ability and desired biocontrol attributes which include broad host range and ability of biocontrol pre and/or post infection through QQ approach.

## Supporting Information

S1 Fig Quantification of C6HSL using agar slice method. In sterile Luria agar (LA) plates, agar was aseptically cut into separated bars of 1cm width by removing 2 to 3 mm slices of agar between the bars. In the agar bars 3mm diameter wells were made. Overnight grown *C*. *violaceum* CV026 was streaked below each well. 6μl of C6HSL (60, 120, 180, 240, 300, 600 picomol) were added to the well and the plates were incubated at static condition for 48 hours at 30°C. The bioassay plates were examined for the presence of purple colour and the distance from the well up to the purple coloration was measured. *C*. *violaceum* CV026 colonies turned purple till it could encounter C6HSL and this distance was proportional to the amount of C6HSL introduced in the well.
(TIF)

S2 Fig Degradation of different chain length AHLs by *Lysinibacillus* sp. Gs50 and *E*.*coli* BL21(DE3) pET22b(+)/*adeH*. A (1, 2, 3) *Lysinibacillus* sp. Gs50+C4HSL, B (1, 2, 3) *E*.*coli* BL21(DE3)pET22b(+)/*adeH* +C4HSL, C (1, 2, 3) C4HSL+ *C*. *violaceum* CV026, D (1, 2, 3) *Lysinibacillus* sp. Gs50+C6HSL, E (1, 2, 3) *E*.*coli* BL21(DE3)pET22b(+)/*adeH* +C6HSL, F (1, 2, 3) C6HSL+ *C*. *violaceum* CV026, G (1, 2, 3) *Lysinibacillus* sp. Gs50+3OC6HSL, H (1, 2, 3) *E*.*coli* BL21(DE3)pET22b(+)/*adeH*+3OC6HSL, A (4, 5, 6) 3OC6HSL+ *C*. *violaceum* CV026, B (4, 5, 6) *Lysinibacillus* sp. Gs50+C8HSL, C (4, 5, 6) *E*.*coli* BL21(DE3)pET22b(+)/+C8HSL, D (4, 5, 6) C8HSL+ *C*. *violaceum* CV026, E (4, 5, 6) *Lysinibacillus* sp. Gs50+ 3OC8HSL, F (4, 5, 6) *E*.*coli* BL21(DE3)pET22b(+)/*adeH*+ 3OC8HSL, G (4, 5, 6) 3OC8HSL+ *C*. *violaceum* CV026, H (4, 5, 6) only *C*. *violaceum* CV026.
(TIF)

S3 Fig Effect of pH on AdeH activity. C6HSL depicts the only substrate at different pH and AdeH + C6HSL depicts enzyme substrate reaction at different pH.
(TIF)

S4 Fig AHL degradation kinetics of AdeH according to Michaelis-Menten equation at 35°C and pH 8.0. (TIF)

S5 Fig Lysis of *Lysinibacillus* sp. Gs50 cells after sonication. Microscopic images of before sonication and after sonication samples stained with (i) Crystal violet and (ii) Combination of trypan blue and nigrosin stains.
(TIF)
