# Supplementary figures and images for: Attenuation of Quorum Sensing Regulated Virulence of Pectobacterium carotovorum subsp. carotovorum through an AHL Lactonase Produced by Lysinibacillus sp. Gs50
Source: PLoS One. 2016 Dec 2;11(12):e0167344. doi: 10.1371/journal.pone.0167344 (PMC5135098; doi:10.1371/journal.pone.0167344)

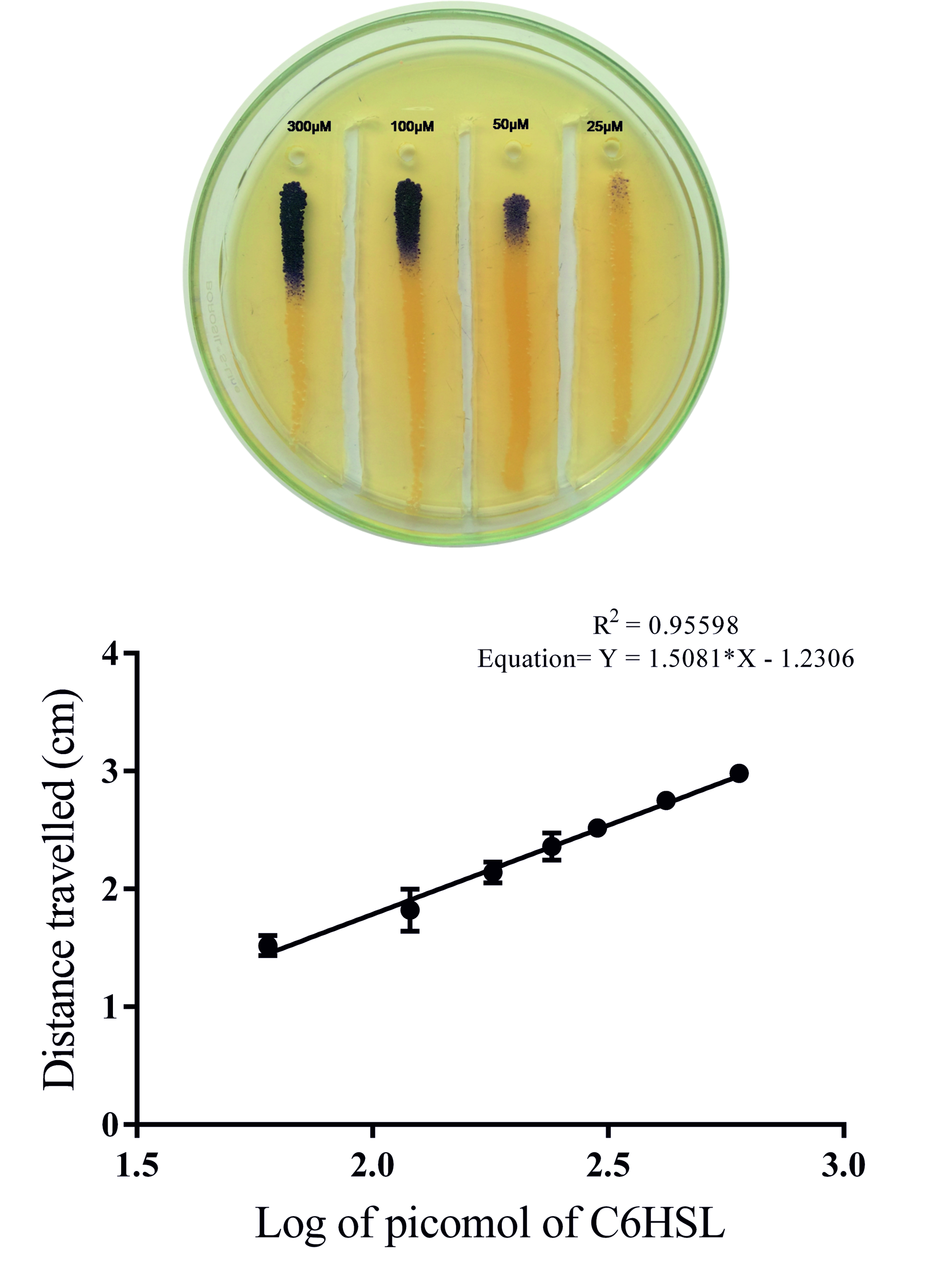

Supplement: S1 Fig — In sterile Luria agar (LA) plates, agar was aseptically cut into separated bars of 1cm width by removing 2 to 3 mm slices of agar between the bars. In the agar bars 3mm diameter wells were made. Overnight grown C. violaceum CV026 was streaked below each well. 6μl of C6HSL (60, 120, 180, 240, 300, 600 picomol) were added to the well and the plates were incubated at static condition for 48 hours at 30°C. The bioassay plates were examined for the presence of purple colour and the distance from the well up to the purple coloration was measured. C. violaceum CV026 colonies turned purple till it could encounter C6HSL and this distance was proportional to the amount of C6HSL introduced in the well. (TIF) [file pone.0167344.s001.tif]

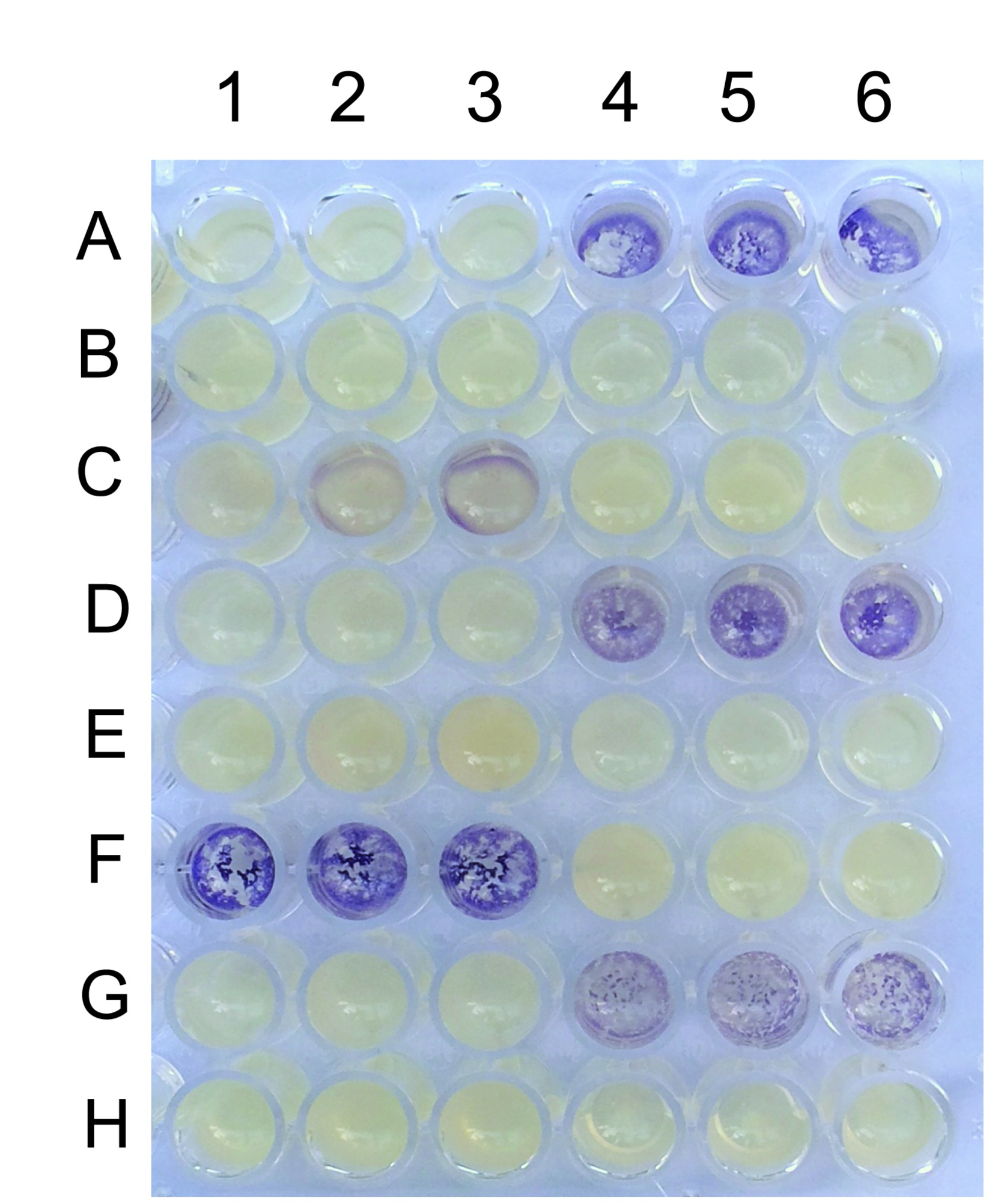

Supplement: S2 Fig — A (1, 2, 3) Lysinibacillus sp. Gs50+C4HSL, B (1, 2, 3) E.coli BL21(DE3)pET22b(+)/adeH +C4HSL, C (1, 2, 3) C4HSL+ C. violaceum CV026, D (1, 2, 3) Lysinibacillus sp. Gs50+C6HSL, E (1, 2, 3) E.coli BL21(DE3)pET22b(+)/adeH +C6HSL, F (1, 2, 3) C6HSL+ C. violaceum CV026, G (1, 2, 3) Lysinibacillus sp. Gs50+3OC6HSL, H (1, 2, 3) E.coli BL21(DE3)pET22b(+)/adeH+3OC6HSL, A (4, 5, 6) 3OC6HSL+ C. violaceum CV026, B (4, 5, 6) Lysinibacillus sp. Gs50+C8HSL, C (4, 5, 6) E.coli BL21(DE3)pET22b(+)/+C8HSL, D (4, 5, 6) C8HSL+ C. violaceum CV026, E (4, 5, 6) Lysinibacillus sp. Gs50+ 3OC8HSL, F (4, 5, 6) E.coli BL21(DE3)pET22b(+)/adeH+ 3OC8HSL, G (4, 5, 6) 3OC8HSL+ C. violaceum CV026, H (4, 5, 6) only C. violaceum CV026. (TIF) [file pone.0167344.s002.tif]

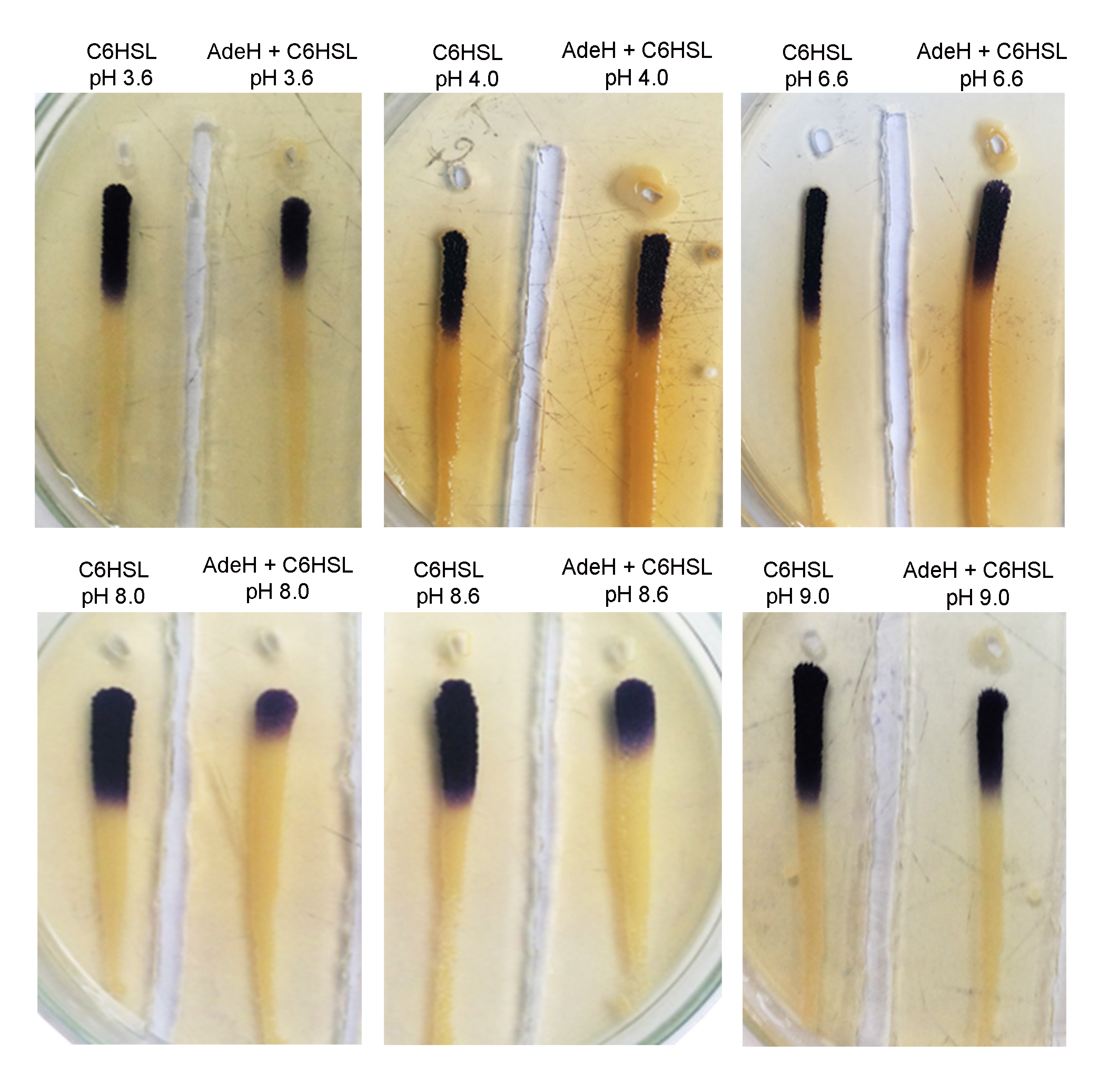

Supplement: S3 Fig — C6HSL depicts the only substrate at different pH and AdeH + C6HSL depicts enzyme substrate reaction at different pH. (TIF) [file pone.0167344.s003.tif]

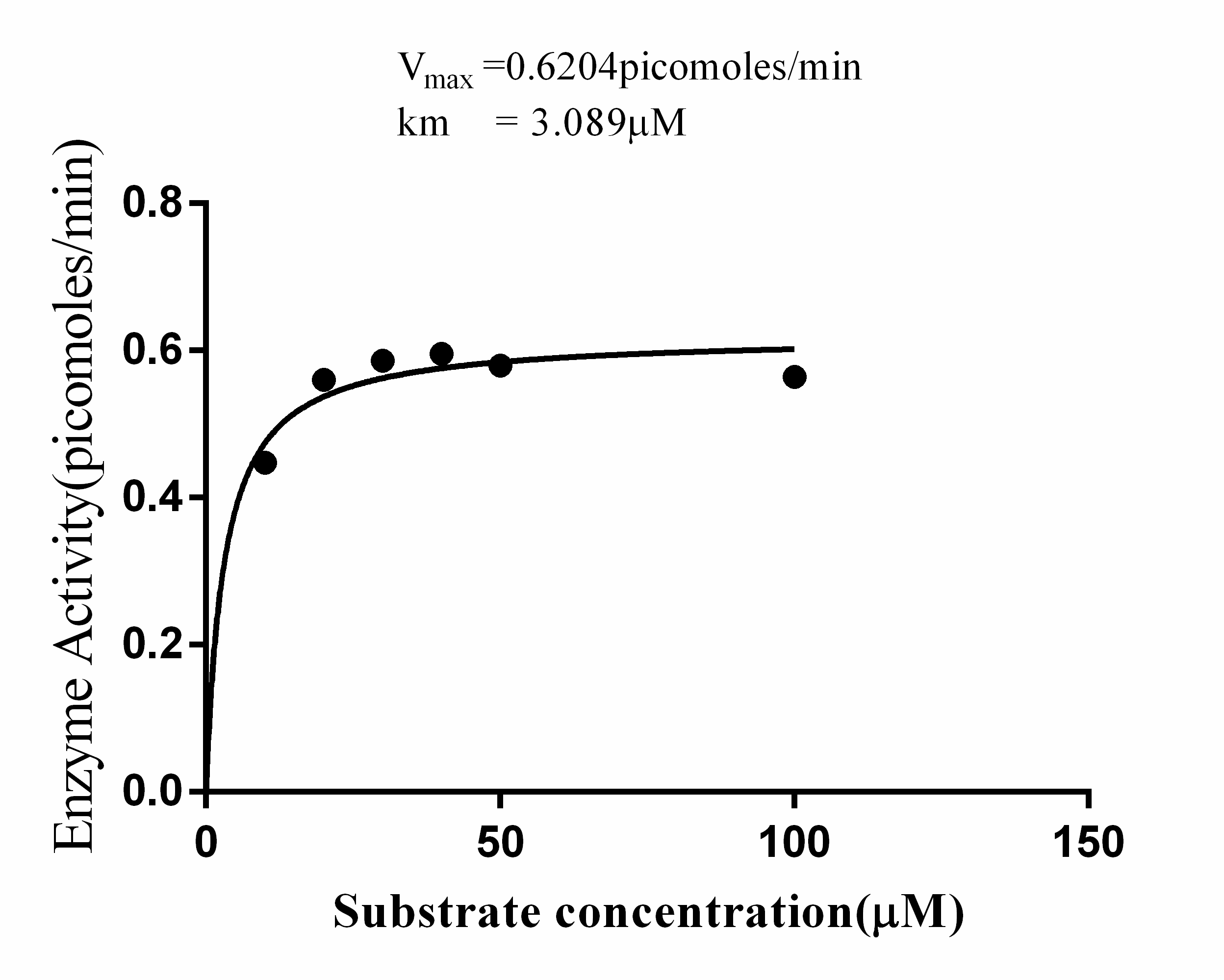

Supplement: S4 Fig — (TIF) [file pone.0167344.s004.tif]

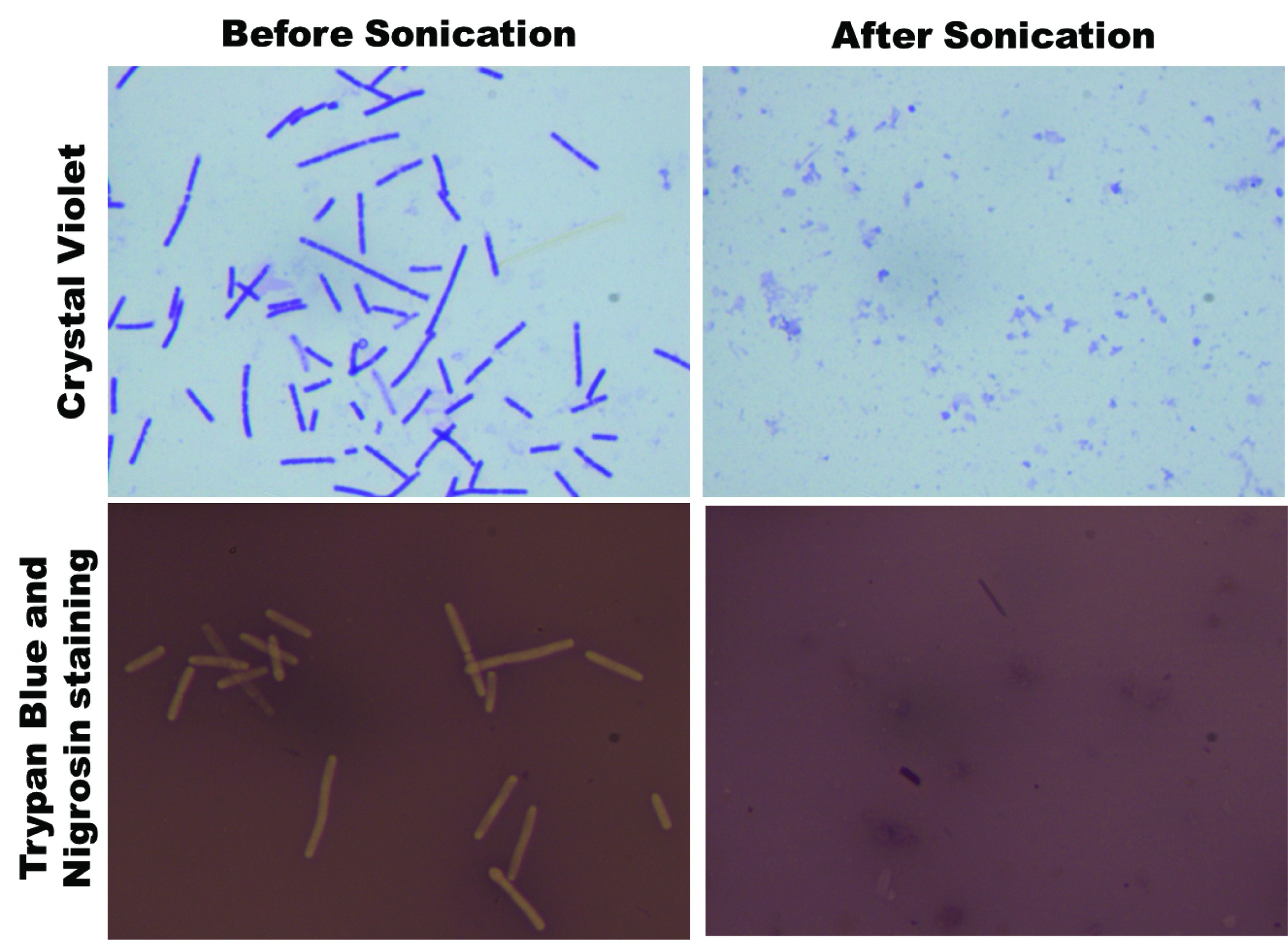

Supplement: S5 Fig — Microscopic images of before sonication and after sonication samples stained with (i) Crystal violet and (ii) Combination of trypan blue and nigrosin stains. (TIF) [file pone.0167344.s005.tif]
